# Synaptic Secretion and Beyond: Targeting Synapse and Neurotransmitters to Treat Neurodegenerative Diseases

**DOI:** 10.1155/2022/9176923

**Published:** 2022-07-25

**Authors:** Ziqing Wei, Mingze Wei, Xiaoyu Yang, Yuming Xu, Siqi Gao, Kaidi Ren

**Affiliations:** ^1^Department of Neurology, The First Affiliated Hospital of Zhengzhou University, Zhengzhou University, Zhengzhou 450052, China; ^2^Henan Key Laboratory of Cerebrovascular Diseases, The First Affiliated Hospital of Zhengzhou University, Zhengzhou University, Zhengzhou 450052, China; ^3^Clinical Systems Biology Laboratories, The First Affiliated Hospital of Zhengzhou University, Zhengzhou 450052, China; ^4^The Second Clinical Medical College, Harbin Medical University, Harbin 150081, China; ^5^The Second Affiliated Hospital of Zhengzhou University, Zhengzhou 450014, China; ^6^Institute of Microcirculation, Hebei North University, Zhangjiakou 075000, China; ^7^Department of Pharmacy, The First Affiliated Hospital of Zhengzhou University, Zhengzhou 450052, China; ^8^Henan Key Laboratory of Precision Clinical Pharmacy, Zhengzhou University, Zhengzhou 450052, China

## Abstract

The nervous system is important, because it regulates the physiological function of the body. Neurons are the most basic structural and functional unit of the nervous system. The synapse is an asymmetric structure that is important for neuronal function. The chemical transmission mode of the synapse is realized through neurotransmitters and electrical processes. Based on vesicle transport, the abnormal information transmission process in the synapse can lead to a series of neurorelated diseases. Numerous proteins and complexes that regulate the process of vesicle transport, such as SNARE proteins, Munc18-1, and Synaptotagmin-1, have been identified. Their regulation of synaptic vesicle secretion is complicated and delicate, and their defects can lead to a series of neurodegenerative diseases. This review will discuss the structure and functions of vesicle-based synapses and their roles in neurons. Furthermore, we will analyze neurotransmitter and synaptic functions in neurodegenerative diseases and discuss the potential of using related drugs in their treatment.

## 1. Background

The nervous system plays an important role in regulating the physiological function of the body [1, 2]; neurons are the most basic structural and functional units of this system [1, 2]. Billions of neurons exist in the nervous system, most of which are distributed in the central nervous system (CNS) of the brain [3, 4]. Neurons can contact each other and transmit information; they use synapse as the site of information exchange, which then determines the function of the nervous system [5]. The synapse is an asymmetric structure composed of presynaptic membrane, postsynaptic membrane, and synaptic cleft between two membranes [6, 7]. Synapse formation involves many extracellular factors, cell adhesion molecules, and intracellular signaling or structural proteins [7]. After the establishment of synaptic connections, synapses undergo structural or functional changes, known as synaptic plasticity [8], which is mediated by neuronal activity and a variety of secreted factors [8].

There is a highly specialized site at the presynaptic nerve terminal, known as the active zone, which is exquisitely designed to facilitate the fusion of synaptic vesicles with the plasma membrane [9, 10]. A high-density region also exists in the postsynaptic membrane [11], which is a protein-rich collection, and is composed of large scaffold proteins, some neurotransmitter receptor proteins, and related elements regulating synaptic activity to form postsynaptic density (PSD) [12, 13], which is the structural basis of postsynaptic signal transduction and integration [14, 15].

The chemical transmission mode of the synapse is realized through neurotransmitters and electrical processes [16, 17]. When the electrical signal transmitted from the cell body reaches the axon terminal, it causes the depolarization of the presynaptic membrane, activates the voltage sensitive calcium channel on the presynaptic membrane, leads to the influx of extracellular Ca^2+^, and subsequently triggers the fusion of synaptic vesicles and presynaptic membrane [18]; then, it releases neurotransmitters into the synaptic cleft [16]. Neurotransmitters in the synaptic cleft bind to specific receptors on the postsynaptic membrane, causing the next neuron or effector cell to complete the signal transmission of the nervous system [19]. According to the differences between chemical transmitters and specific receptors, postsynaptic potentials can be classified into two types [20–23], as follows: excitatory postsynaptic potential (EPSP), which depolarizes the postsynaptic membrane and manifests as the excitation of postsynaptic neurons [23], and inhibitory postsynaptic potential (IPSP), which hyperpolarizes the postsynaptic membrane and manifests as the inhibition of postsynaptic membrane excitability [22]. Neurotransmitters can only be released into the synaptic cleft through vesicles in the presynaptic membrane to act on the postsynaptic membrane [16, 17]. The unidirectionality of chemical synapses [16, 17], the specificity of postsynaptic receptors [24], and the plasticity of chemical synapses ensure that the postsynaptic membrane selectively receives and transmits the information in an orderly manner from the presynaptic membrane [25, 26], based on the fact that synapse is a functional unit of the brain, whose dysfunction can lead to a series of neurorelated diseases [27–29].

## 2. Basic Process of Synaptic Secretion

**Figure 1:**
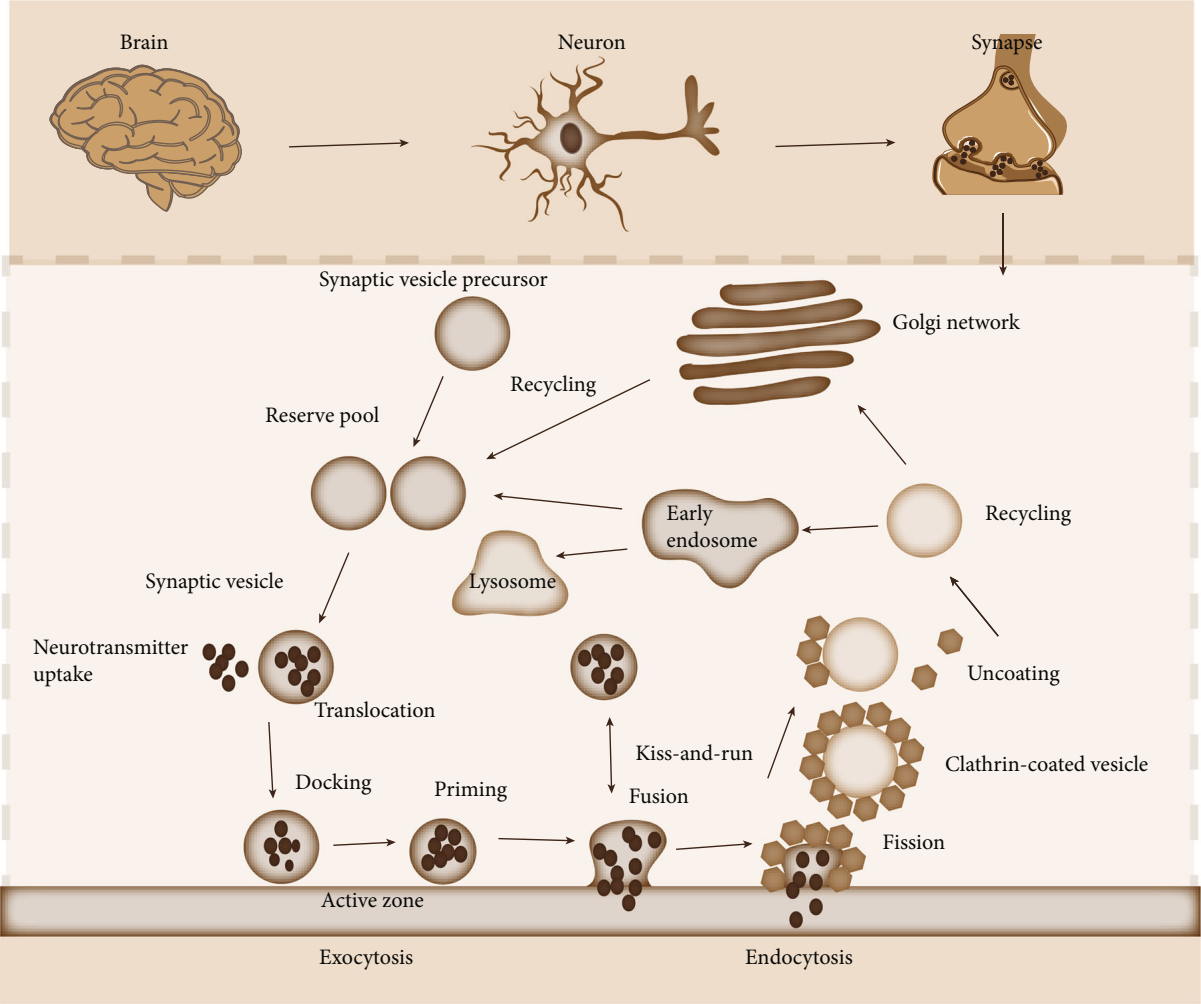
Secretory process and recycling of synaptic vesicles. The synaptic vesicle cycle consists of exocytosis, endocytosis, and recycling. Synaptic vesicles filled with neurotransmitters are docked to the presynaptic active zone by translocation, where the vesicles undergo a priming reaction. When they fuse with the presynaptic membrane, the neurotransmitters are released. Subsequently, synaptic vesicles undergo endocytosis and recycling.

Synapses communicate with one another by releasing neurotransmitters and other chemicals from presynaptic vesicles [30, 31]. Vesicles are widely reported as among the important functional structural components of the endomembrane system that are directly transported to different membrane structures [32, 33]. According to the different morphologies and contents, two kinds of vesicles were involved in exocytosis, namely, small clear vesicles (SCVs) and dense core vesicles (DCVs) [34, 35]. SCVs become synaptic vesicles (SVs) at the end of neurons [36]. In mammals, the diameter of SVs is generally less than 50 nm, and the vesicle contents are small molecular neurotransmitters, such as acetylcholine [35]. DCVs are distributed in the axons and dendrites of neurons and have diameters in the range of 70–200 nm [37]. The DCVs' contents include neuropeptides, nerve growth factor, monoamine, and other neuromodulatory substances [35, 37]. Although morphological structure and function differ between the two kinds of vesicles, the exocytosis processes of vesicles are the same, including tethering, docking, priming, and fusion [38] (Figure 1). Neurotransmitter secretion is the fusion process of synaptic vesicle and presynaptic membrane and is a calcium-dependent process (Figure 1) [19, 39].

The increase of intracellular calcium concentration triggers the fusion between synaptic vesicles and presynaptic membrane, resulting in the release of neurotransmitters [39–41]. During the fusion, a hydrophilic pore called fusion pore is formed [42–44]. Chemicals in vesicles need to be released through fusion pores [42–44]. Vesicle fusion is an energy-consuming process, and the zipper assembly of Soluble N-ethylmaleimide-Sensitive factor Attachment protein REceptor (SNARE) complex can provide energy for membrane fusion [45, 46].

The contents of vesicles are believed to be released through two main modes [43, 47, 48]. One mode is the incomplete fusion and rapid closure (kiss-and-run) that limits the release of substances in vesicles [49–51]. This mode only allows catecholamines and other small molecules to be released through a narrow fusion pore [49–51]. The other mode is the irreversible expansion of the vesicle membrane until it flattens (full collapse) to promote the complete fusion of transmitter release [52–54]. Studies have found that both full collapse and kiss-and-run modes exist simultaneously in the CNS, and the two modes can be interchanged to better complete the vesicle recycling cycle [52, 55]. In the intimal fusion system, besides the transporting of neurotransmitters and other substances to the plasma membrane through vesicles and releasing them to the synaptic cleft through membrane fusion in exocytosis [56–58], endocytosis is also required to recover extracellular molecules into the cell to supplement raw materials [59], such as lipids or proteins, for the next round of intracellular activities [60, 61]. This series of complex biological reactions constitutes a dynamic and efficient membrane fusion system [57, 58, 62–64]. Numerous proteins and complexes that are widely reported to regulate these processes have been identified, and their regulation of synaptic vesicle secretion is complicated and delicate [57, 62].

## 3. Regulatory Proteins and Mechanisms in Synaptic Secretion

Three decades of researches and many major discoveries have been reported, providing important insights into synaptic secretion and generating a functional model of Ca^2+^-triggered neurotransmitter release mechanisms mediated by protein-protein interaction cascades with SNARE complex as the core [65].

### 3.1. SNARE Proteins

Soluble N-ethylmaleimide-Sensitive factor Attachment protein REceptors (SNAREs) are a molecular machine that mediate such membrane fusion [45, 46]. SNAREs have been identified and elucidated in *Saccharomyces cerevisiae* over the past few decades [66]. In fungi, more than twenty subtypes of SNARE proteins exist and function in different organelles or cellular regions [66, 67]. In multicellular organisms, the number of SNARE subtypes varies from 30 to 50 [68, 69]. Notably, in the nervous system, SNARE complexes are composed of three proteins [45, 70]; the canonical and most well-defined SNAREs are as follows: syntaxin-1 and SNAP-25 (synaptosome-associated protein of 25 kDa) located in the presynaptic membrane, which belong to t-SNARE (target-SNARE) [45]; VAMP-2/Synaptobrevin-2 (vesicle-associated membrane protein) located in the membrane of synaptic vesicles, which belongs to v-SNARE (vesicle-SNARE) [71]. Syntaxin-1 and VAMP-2/Synaptobrevin-2 are anchored to the presynaptic membrane and synaptic vesicle membrane via the C-terminal transmembrane region, respectively [66], whereas SNAP-25 has no transmembrane region and is anchored to the presynaptic membrane via the fatty acyl group of four cysteine residues in the mesenchymal region [72].

SNARE complexes are formed by binding to each other through SNARE motifs [73–75]. Although SNARE proteins differ in amino acid length and structure, the SNARE motifs with a length of about 65 amino acids are highly conserved [76, 77]. When SNARE proteins exist alone, their SNARE motifs are mostly random curls; when these regions are combined together, they fold to form tight SNARE core complexes [78, 79]. The crystal structure of the core complex consists of parallel four helical bundles with an overall length of 12 nm [79]. Among the helical bundles, both syntaxin-1 and VAMP-2/Synaptobrevin-2 provide one *α*-helix, and SNAP-25 provides two *α*-spirals [80]. The core of the helix bundle consists of 15 layers of hydrophobic amino acid residues, except for the layer called “0” in the center of the helix bundle [81], which is a hydrophilic layer containing one arginine residue and three glutamine residues that form hydrogen bonds within the hydrophobic core [79]. Arginine residues come from VAMP-2/Synaptobrevin-2 and are called R-SNARE proteins [82]. Three glutamine residues come from syntaxin-1 and SNAP-25 and are named Q-SNARE proteins [82]. Among them, syntaxin-1 is called Qa, and the N-terminal and C-terminal of SNAP-25 protein are called Qb and Qc, respectively [82]. Biochemical experiments showed that the SNARE core complex has high thermal stability [46, 83, 84]. These characteristics show that the formation of the complex is very favorable in terms of energy, which is a key feature of current membrane fusion models [85, 86].

In the process of vesicle fusion, the assembly of SNARE complex is ordered from N- to C-terminal, also known as N-terminal nucleation [87–90]. The assembly energy of each layer of SNARE complex differs. Macroscopically, the energy released by N-terminal (-7 layers to -1 layer) assembly is higher than that of C-terminal (+1 to +8 layers) and also more stable after installation [81, 91]. The C-terminal assembly is reversible, because the N-terminal contains more hydrophobic amino acids with larger side chain volume compared with the carbon end, thereby providing more binding energy and making the hydrophobic core closer [85, 90]. Considering that the N-terminal and C-terminal of SNARE complex have different thermodynamic properties [88], the assembly of SNARE complex is a stepwise process; that is, the N-terminal is responsible for nucleation effect and stable assembly state, whereas the C-terminal connects the assembly of the SNARE complex with membrane fusion process [88, 89, 92]. Although this theory is deeply supported by theory and experiment, several studies demonstrated that the assembly of the SNARE complex *in vitro* is continuous [92].

According to previous studies, the energy released by the assembly of SNARE complexes is close to 35 *k*_B_*T*, and this energy is enough to overcome the barrier and lead to fusion [90, 93], which means that only one group of SNARE complexes can complete the membrane fusion process [94]. In fact, the conclusion is based on the continuous assembly of SNARE complex [94]. However, there are multiple proteins and complexes regulating the assembly of SNARE complex under physiological conditions [95]. The assembly of SNARE complex is unlikely to meet the conditions of continuous assembly under precise regulation [96, 97]. Therefore, five to six groups of SNARE complexes are needed to meet the formation of fusion pores between synaptic vesicles and presynaptic membranes [96, 97].

### 3.2. Munc18-1

Munc18-1 is a member of the Sec1/Munc18 (SM) protein family [98–100], which is expressed in neurons and neuroendocrine cells and plays an important role in the release of neurotransmitters [101–104]. Multiple experimental evidences show that Munc18-1 is involved in the process of synaptic vesicle anchoring, priming, and fusion [105–107]. These functions are related to the interaction between Munc18-1 and SNARE proteins [108], the most significant of which is syntaxin-1 [104, 105, 108, 109]. The interaction surface between Munc18-1 and syntaxin-1 is complicated, and the binding modes are diverse, which is why the affinity between Munc18-1 and syntaxin-1 is high [105, 110].

The binding of Munc18-1 to syntaxin-1 is important for the regulation of synaptic vesicle secretion [107, 111]. The kinetic data show that free syntaxin-1 exists in a mixture of at least two different conformations [112]. When syntaxin-1 combines with Munc18-1, Munc18-1 can make syntaxin-1 in a stable closed conformation [38, 109, 113]. In addition, Munc18-1, as a molecular chaperone, contributes to the correct transport and localization of syntaxin-1 [102, 109, 114]. Munc18-1/syntaxin-1 complex can prevent syntaxin-1 from forming a heterodimer with SNAP-25, affect the formation of normal SNARE complex, and protect syntaxin-1 before the arrival of the signal [113, 115].

Although the combination of Munc18-1 and syntaxin-1 is also important for the fusion of vesicles, the results of SNARE complex recombination experiment *in vitro* show that when syntaxin-1 exists as Munc18-1/syntaxin-1 complex, the SNARE motif H3 of syntaxin-1 is locked and cannot participate in the formation of SNARE complex, resulting in the incomplete vesicle fusion [105, 113]. The results *in vitro* seem to contradict the physiological results *in vivo*; however, the contradiction is resolved with the analysis of the function of the regulatory factor Munc13-1 [116, 117]. The recombination experiment *in vitro* showed that Munc13-1 could change Munc18-1/syntaxin-1 complex from “closed” state to “open” state, thereby forming the SNARE complex [117–119]. Therefore, Munc18-1 initiates the assembly of SNARE complex and ultimately achieves the fusion of vesicles [106, 107, 120].

### 3.3. Synaptotagmin-1

The speed of information transmission by the nervous system can be accounted by millisecond and depends on calcium signals [57]. In the presynaptic membrane region, a calcium receptor that can respond to calcium signal called Synaptotagmin-1 is present [121–123]. Synaptotagmin-1 is anchored to synaptic vesicles by its N-terminal transmembrane domain [124]. The cytoplasmic region of Synaptotagmin-1 contains two C_2_ domains, which are called C_2_A and C_2_B [123, 124]. C_2_A binds three calcium ions, whereas C_2_B binds two calcium ions [122, 125, 126].

In response to calcium ions, the two C_2_ domains of Synaptotagmin-1 bind to negatively charged biofilms and shorten the distance between synaptic vesicles and the presynaptic membrane [124, 125]. Thus, they reduce the energy barrier to be overcome and ultimately mediate the fusion of synaptic vesicles and the release of neurotransmitters by presynaptic membrane [57]. The C_2_B domain of Synaptotagmin-1 has two specialized regions that are rich in basic amino acids [127, 128]. One region is called the polybasic stretch, which consists of two amino acid sites, namely, K326 and K327 [129]. The other region is called R398-399 [130], which consists of two positively charged amino acids, namely, R398 and R399. These two regions bind phosphatidylinositol-4, 5-diphosphate (PIP2) and SNARE complexes enriched in the presynaptic membrane, respectively, which are particularly important for the function of Synaptotagmin-1 [129, 130]. They work together to close the distance between vesicles and the presynaptic membrane, stabilize vesicles anchoring or initiating in the presynaptic active region, and prevent the further assembly of SNARE complexes [128, 131].

At this point, Ca^2+^ in the C_2_ domain binds to the pocket's negatively charged amino acid residues and targets the membrane to generate a same-charge repulsion, thus inhibiting the fusion process of synaptic secretion [131]. After Ca^2+^ influx, Ca^2+^ binds the pocket of the C_2_A domain and C_2_B domain and thereby shields the negative charge and results in a net positive charge [125, 132]. This positive charge and the positive charge of the highly conserved amino acid residues on each pocket act like an instantaneous electrostatic switch, pulling vesicles closer to the negatively charged presynaptic membrane [133]. Meanwhile, the insertion of pocket hydrophobic amino acid residues in the C_2_ domain causes lipid disorder, changes the membrane curvature, and deforms the membrane [134], which is conducive to the transformation of the trans-SNARE complex to the cis-SNARE complex and ultimately promotes membrane fusion and neurotransmitter release [135–138].

### 3.4. Munc13-1

Munc13-1 contains three C_2_ domains, namely, C_2_A, C_2_B, and C_2_C domains [139]. The C2A domain can interact with the upstream of the Rab3-interacting molecules (RIMs) [140]. RIMs are a class of Rab3 effectors with high molecular weight and exist as scaffold proteins of the active zone in the presynaptic membrane [135, 141]. The C_2_B domain is the only one of the three C_2_ domains in Munc13-1 that can bind Ca^2+^ and PIP2 [142]. The C_2_B domain of Munc13-1 can be used as a potential calcium receptor. A C_1_ domain, which can bind diacylglycerol (DAG), is present at the N-terminal of the C_2_B domain [143–145]. The combination of C_1_ and C_2_B domains enables Munc13-1 to bind phospholipid molecules in the presynaptic membrane [146]. The C_2_C domain at the C-terminal does not bind Ca^2+^ and negatively charged phospholipid molecules in the presence of Ca^2+^ [144]. However, it can bind to the fatty acid chain inside the phospholipid bilayer due to the existence of hydrophobic amino acids in its periphery so that the C_2_C domain can nonselectively have affinities to the membrane [147]. A calmodulin-binding motif (CaMb) also exists near the N-terminal of the C_1_ domain [148, 149], which is believed to be strongly correlated with the function of Munc13-1 in calcium-regulated neurotransmitter secretion [150–154].

The most important core domain of Munc13-1 protein is the central MUN domain [155]. The MUN domain, as a key functional element of Munc13, plays an important role in synaptic secretion [155]. MUN domain is also present in BAP3, CAPS, and other proteins in most eukaryotes [156]; it is structurally similar to other CATCHR family members that play roles in different transport steps [157, 158]. These CATCHR proteins form a series of aligned *α*-helical bundles with flexible hinge regions that bind vesicles to the fusion sites, suggesting that Munc13 can participate in the process of vesicle binding through the MUN domain [158, 159]. In addition, the MUN domain of Munc13-1 interacts weakly with SNARE complexes, Munc18-1, and SNARE motif of syntaxin-1, which are essential for Munc13-1 function [116, 155, 160–163].

Munc13-1 is also involved in the opening of the syntaxin-1 protein closed by Munc18-1 [117, 119] and can significantly accelerate the transformation of syntaxin-1 from Munc18-1/syntaxin-1 complex to SNARE complex depending on the “NF” pocket catalytic active center in MUN domain [118]. Recent studies showed the interaction between VAMP2/Synaptobrevin-2 and Munc13-1 MUN domain and analyzed the crystal structure of this complex [164]. This quaternary complex cooperates to start the assembly and membrane fusion process of the SNARE complex [161, 165, 166]. These studies revealed the function and molecular mechanism of Munc13-1 in SNARE complex assembly and synaptic vesicle priming, thereby providing a strong theoretical basis for understanding the molecular mechanism of neural signal transduction [165, 167].

### 3.5. CAPS-1

Mammals express two CAPS isoforms, namely, CAPS-1 and CAPS-2, which are in neurons and endocrine cells [168]. CAPS is a multidomain protein that contains the following: the C_2_ domain, which is involved in CAPS dimerization [169, 170]; pleckstrin homologous (PH) domain, which is characterized as a PIP2-binding domain to mediate CAPS interaction with the plasma membrane [171–176]; DAMH domain, which exhibits sequence homology to the Munc13 MUN domain and is required for CAPS binding to SNAREs [156, 158, 171, 177–179]; and dense core vesicle binding domain (DCVBD), which appears to be important for CAPS' association with DCVs [172, 180].

Both CAPS-1 and Munc13-1 contain key regions that bind to PIP2 clusters on the plasma membrane, but unlike Munc13-1, CAPS-1 binds PIP2 through the PH domain in a calcium-independent manner [171, 173]. CAPS and Munc13 are both the promoters of DCVs and SVs exocytosis, and their functions are nonredundant [181–184], whereas the molecular mechanism underlying the regulation of exocytosis secretion in time and space has not been clarified.

Interestingly, a study showed that natural CAPS-2 splicing isomer, which has C_2_-PH domains and misses DAMH and DCV binding domains, can rescue the exocytosis of chromaffin cells and neurons lacking CAPS-1 and CAPS-2 [185]. This activity increases the possibility that the initiation of DCVs in the early development stage of chromatin cells can be realized through the C_2_-PH domain, whereas the initiation function of CAPS needs the participation of other domains in more mature cells [185]. A subsequent study about the successful crystal structure analysis of the DAMH domain offers the possibility of further understanding the function of CAPS-1, thereby revealing the dual role of CAPS-1 in SNARE complex formation [186], as follows: (1) CAPS-1 DAMH domain interacts with Munc13-1 MUN, and the interaction hinders Munc13-1 activity to open Munc18-1/SNARE, which further leads to the assembly of the SNARE complex failure. (2) After syntaxin-1 is activated, CAPS-1 stabilizes the active state of syntaxin-1 through the interaction between the DAMH domain with the syntaxin-1/SNAP25 complex, thereby accelerating the assembly of the SNARE complex and finally promoting synaptic exocytosis [178].

**Figure 2:**
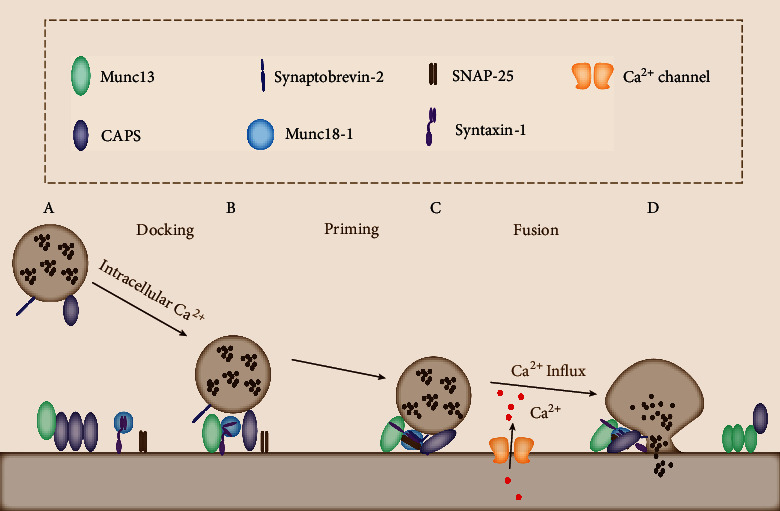
The working model of CAPS–Munc13 in vesicle exocytosis. (A) In the resting state, CAPS-1 is first located on the cytoplasmic membrane; Munc13-1 cannot bind to Munc18-1/syntaxin-1 complex, resulting in the anchored DCVs and the inability of SVs to enter the vesicle priming stage. (B) Under the action of intracellular Ca^2+^, Munc13-1 protein that successfully escapes the inhibition of CAPS-1 can bind to Munc18-1/syntaxin-1 complex and catalyze the opening of syntaxin-1. (C) When the syntaxin-1 protein is open and SNAP-25 exists, CAPS-1 binds to syntaxin-1/SNAP-25 complex to further stabilize the open state of syntaxin-1 then promotes binding with Synaptobrevin-2 to form the SNARE complex. (D) With the influx of extracellular Ca^2+^, vesicle membrane fusion can occur quickly and effectively; then, the release of neurotransmitters occurs.

Therefore, based on these studies, a model in which CAPS and Munc13 jointly regulate vesicle secretion was proposed (Figure 2) [186]: in the resting state, CAPS-1 is first located on the cytoplasmic membrane through the calcium-independent interaction between PH and PIP2. Munc13-1 cannot bind to Munc18-1/syntaxin-1 complex due to the interaction of PH–PIP2 and DAMH–MUN [173, 186]. Thus, the anchored DCVs and SVs cannot enter the vesicle priming stage. Under the action of intracellular calcium level, CAPS-1 and Munc13-1 can promote vesicle recruitment to the PIP2-rich cytoplasmic membrane in a calcium-dependent manner. At this time, some Munc13-1 successfully escape the binding and inhibition of CAPS-1 protein; then, Munc13-1 can bind to Munc18-1/syntaxin-1 complex and catalyze the opening of syntaxin-1. When syntaxin-1 protein is open and SNAP-25 exists, CAPS-1 binds to syntaxin-1/SNAP-25 complex to further stabilize the open state of syntaxin-1 and promotes binding with Synaptobrevin-2 to form the SNARE complex [178]. With the influx of extracellular calcium, the increase of intracellular calcium level will activate phospholipase PLC*η*2 that leads to PIP2 hydrolysis and DAG formation [181].

Subsequently, the hydrolysis of PIP2 will lead to the decrease of CAPS-1 activity, and the increase of DAG level will stabilize the function of Munc13-1 protein [181, 182]. Some key fusion proteins, including complexin-1 and Synaptotagmin-1, may also promote the formation of the SNARE complex together with CAPS-1 and Munc13-1 so that vesicle membrane fusion can occur quickly and effectively [97, 187]. Although this model needs to be further improved and clarified, it demonstrates a strong sequence and coordination between CAPS-1 and Munc13-1 in the formation of SNARE complexes; it also shows that the calcium-dependent spatial distribution of PIP2 and DAG changes the distribution of CAPS-1 and Munc13-1 in the presynaptic membrane and modulates their activity [186].

In addition to the proteins described above, there are a large number of Ca^2+^ channels in presynaptic nerve terminals to regulate the concentration of Ca^2+^ in neurons ([Ca^2+^]i), which play important roles in the release of neurotransmitters [188–190]. There are many types of Ca^2+^ channels with different molecular compositions and properties [188], which are mainly P/Q-type and N-type channels (referred to as Ca_v_2.1 and Ca_v_2.2) responsible for initiating synaptic transmission at fast conventional synapses [189, 191, 192]. These Ca^2+^ channels coexist in the same presynaptic nerve terminals and have a synergistic relationship to promote transmitter release [193]. The inhibition of the activity of any type of Ca^2+^ channel will reduce the release of presynaptic transmitter. The plasma membrane SNARE proteins (syntaxin-1 and SNAP-25) and synaptotagmin-1 can specifically interact with the channels in a Ca^2+^-dependent manner by binding to the synaptic protein interaction (*synprint*) sites of Ca_V_2.1 and Ca_V_2.2 channels [194–196]. This interaction regulates channel function and thus controls synaptic transmission [197].

## 4. Neurotransmitters and Synaptic Function in Neurodegenerative Diseases

Synapses are the functional part of the connection between neurons and the key part of the physiological function of neurons [8, 198]. They are not in a static state in the body and undergo relatively lasting dynamic changes called synaptic plasticity under the stimulation of neuronal activity or other factors [8, 31, 199]. Changes in synaptic plasticity are the main mechanisms of the CNS growth, development, learning, and memory [8, 199]. Degenerative alterations include loss of synapses, branch atrophy, and cell death in different types of cells, such as cholinergic, glutamatergic, noradrenergic, and inhibitory neurons [200]. In clinical patients or animal models, structural degeneration, such as reduction in neurons, generally does not appear until the middle-late stage, and cognitive impairment in the early stage of the disease is more likely to be caused by abnormal synaptic function in specific brain regions (prefrontal cortex and hippocampus) [201].

In fact, many studies showed that the oligomeric A*β* protein, a characteristic pathological marker of Alzheimer's disease (AD) [202], has strong synaptic toxicity, which specifically reduces synaptic density, damages long-term synaptic enhancement, facilitates long-term synaptic weakening, and suppresses brain learning and memory function [203–205]. The generation or disturbance of neural activity is largely determined by the state of excitation-inhibition balance, which is closely related to the release and circulation of neurotransmitters in the neural circuit [206].

Therefore, exploring changes in the neurotransmitter system is critical to the elucidation of the biochemical mechanisms of normal aging and age-related neurological/psychiatric disorders such as Parkinson's disease (PD), AD, presenile deafness, and depression (Tables 1 and 2). Several types of transmitters are released from the presynaptic neuron in the CNS, such as glutamate, GABA, and dopamine [207], whereas the neurotransmitters released from neuromuscular connectors are acetylcholine [208, 209]. Synaptic changes in the CNS are often the main manifestations and thus turn into important targets in the clinical therapy of neurodegenerative diseases. Presently, pharmacological interventions of cholinergic and glutamatergic neurotransmission, including cholinesterase inhibitors and N-Methyl-D-Aspartate (NMDA) receptors antagonist, are the only FDA-approved medications for AD but are unable to significantly improve cognitive dysfunction [210]. Similarly, treatments of PD are symptomatic, and levodopa is the typical pharmacologic approach, but with limited modifying effects as well [211]. As a consequence, it is of great theoretical and practical value to study the synaptogenesis and pathological changes in the CNS to further elucidate neurodegenerative diseases [212].

### 4.1. Glutamate

Glutamate (Glu) is the most important excitatory neurotransmitter in mammalian CNS [213]. Glu serves multiple functions in the brain, and such functions are mediated by Glu receptors [214, 215]. The activation of Glu receptors is involved in rapid excitatory synaptic transmission and regulates neurotransmitter release, synaptic plasticity, long-term synaptic enhancement, long-term synaptic inhibition, and other normal physiological functions in the CNS [213, 216]. However, high Glu concentration in the intercellular space can produce toxicity to neurons and lead to neuronal degeneration, senescence, and death [217].

The excitatory toxicity of glutamate is closely related to the occurrence and development of many neurodegenerative diseases and is the important mechanism of the death of nerve cells in neurodegenerative diseases [217, 218]. Glu receptors play two main roles in neurodegenerative diseases [214, 215, 218, 219]. One role is to participate in normal synaptic transmission and serve a neuroprotective function when synaptic activity is enhanced [214, 215]. Another role is the excitatory toxicity mediated by ionic Glu receptors [217, 218].

Excitatory toxicity refers to the neurotoxic effects of the overdose of excitatory amino acids (EAA) and involves two mechanisms [220]. One mechanism is mediated by the overexcitation of NMDA receptors, which can occur over hours to days and is characterized by sustained Ca^2+^ influx and delayed injury of nerve cells [221]. Mitochondrial function can therefore be lost due to large influx of Ca^2+^ and the rapid accumulation of Ca^2+^ in mitochondria [222]. The activity of nitric oxide synthase can also increase, so that NO synthase can increase the toxicity of nerve cells [223]. In most pathological cases, delayed injury of nerve cells caused by Ca^2+^ influx and mediated by NMDA receptor overexcitation dominates excitatory toxicity [205, 221]. The other mechanism is mediated by hyperactivation of *α*-Amino-3-Hydroxy-5-Methyl-4-Isoxazolepropionic Acid (AMPA) and KA receptors [224], which can occur within hours and are characterized by Na^+^ influx, passive influx of Cl^−^ and water, and acute osmotic swelling of nerve cells [225]. The normal structure of the glutamatergic system and the function of Glu transporters and reuptake of Glu were altered in the brain tissues of AD patients [226]. In addition, *β* amyloid precursor protein (APP) and tau protein can inhibit extracellular Glu uptake, which leads to increased extracellular Glu levels, resulting in excitotoxic effects [227–229].

In PD patients and experimental animal models, there is a large increase in Glu neurons projecting from the dorsal subthalamic nucleus to the substantia nigra striatum [230, 231]. These studies confirm that the overactivation of Glu receptors on dopamine neurons is one of the causes of excitatory toxic cell death [232, 233]. Meanwhile, Glu uptake disorder also aggravates Glu receptor hyperactivation that leads to excessive calcium influx, which ultimately further leads to nerve cell death and a series of acute or chronic neurodegenerative diseases (such as stroke and AD) [234].

Several drugs are developed for diseases caused by Glu, such as ginsenoside Rb3, which can reduce the increase of Ca^2+^ in neurons possibly by inhibiting calcium influx induced by NMDA receptors and alleviating calcium overload, thereby preventing hypoxic injury caused by cerebral ischemia [235–237]. Huperzine A can inhibit the NMDA-induced toxicity of the cerebral cortex and synaptic plasma membrane [238–240]. In addition, memantine is an antagonist of NMDA receptors and antagonizes excitatory amino acid toxicity to neurons [241–243].

### 4.2. GABA

*γ*-Aminobutyric acid (GABA) is the most widely distributed inhibitory neurotransmitter in the CNS [244]. It is formed by the removal of carboxyl group of Glu in the brain under the action of glutamic acid decarboxylase (GAD) [245]. GABA participates in a variety of metabolic activities and has high physiological activity [244]. Immunological studies show that the highest concentration of GABA is found in the substantia nigra, and at least 70% of the afferents to substantia nigra dopaminergic neurons are GABAergic [246]. The cognitive impairment caused by nervous system diseases, such as severe depression and epilepsy, is directly related to the increase or decrease of GABA transmission [246, 247]. Changes in brain GABA content and receptor function are crucial for many factors of learning and memory [244, 247]. On the one hand, when the content of GABA in the brain is reduced or the receptor function is impaired, it can induce neurological diseases related to cognitive impairment, and appropriate supplementation and repair of GABA function can improve the cognitive impairment [246, 247]. On the other hand, if the excitatory neurons are overexcited, then they will produce excitatory toxicity, which will eventually lead to abnormal activity of the neural network and lead to cognitive deficits [220]. When GABA is activated, it can inhibit the neurotoxic effect caused by Glu abnormal excitation and improve the learning and memory function decline caused by neural abnormalities [207, 248].

GABA receptors are divided into three types, namely, GABA_A_ receptors, GABA_B_ receptors, and GABA_C_ receptors [247]. Different types of GABA receptors distributed in different brain regions have different mechanisms underlying learning and memory [249]; however, they all have inhibitory effects [247]. Their receptor antagonists can improve the inhibitory effect of learning and memory, which may be due to the promotion of the release of excitatory neurotransmitters in the synapses [250]. The neurotransmitters reach a coordinated and balanced state. GABA works in the adult brain primarily by acting on GABA_A_ and GABA_B_ receptors [251].

GABA_A_ receptors can be activated by a high concentration of GABA and are a kind of ligand-gated Cl^−^ channel receptor that induces synaptic inhibitory response [252]. They affect the rhythmic activity generated in the neural network. The application of GABA_A_ receptor antagonist Bicuculline (Bic) can improve the spatial learning and memory disorders caused by propofol (Pro) [250]. GABA_B_ receptors are metabolic G protein-coupled receptors (GPCRs) that regulate synaptic transmission and are involved in multiple brain functions, such as recognition, learning, memory, and anxiety [253, 254]. Experimental studies showed that baclofen, a GABA_B_ receptor agonist, could affect the acquisition and consolidation of learning and memory [255–257]. CGP35348, a GABA_B_ receptor antagonist, can improve this situation, because CGP35348 inhibits the inhibitory postsynaptic electrical potential (IPSP) and enhances the activation of GABA receptors [248], thereby promoting memory formation [258]. The GABA_C_ receptors are similar to the GABA_A_ receptors but are insensitive to Bic and baclofen [259, 260]. The GABA_C_ receptor antagonist TPMPA can block the inhibitory effect of GABA at lower doses on learning and memory [261].

Although the research on the influence of GABA on cognition has achieved certain success and has guided the treatment of clinical cognitive disorders, the specific mechanism underlying the influence of GABA signal on learning and memory has not been fully elucidated and needs further discussion.

### 4.3. Dopamine

Another neurotransmitter associated with disease is dopamine (DA) [262]. DA regulates various physiological functions of the CNS [263, 264]. The dysregulation of DA system affects the progression of PD, schizophrenia, Tourette syndrome, attention deficit hyperactivity syndrome, and pituitary tumor [265]. PD is a slow progressive neurodegenerative disease that affects middle-aged and elderly population [266], and the main pathological change is the progressive death of dopaminergic neurons in the substantia nigra (SN), which eventually leads to the severe loss of DA in the striatum [267, 268]. The formation of Lewy body is one of the main pathological changes of PD [269]. A close connection exists between the DA system and *α*-synuclein, which is the main component of Lewy body [227, 269].

In the process of DA metabolism, the activity of DA-induced intermediates can be inhibited by combining with *α*-synuclein that selectively induces the formation of *α*-synuclein fibrils and increases fibrillary aggregation [270]. Similarly, the abnormal aggregation of *α*-synuclein leads to the imbalance of normal anabolism of DA, the increase of intracellular toxic-free DA, and the blocking of the vesicle transport of DA [270]. This vicious cycle is formed, thereby intensifying the occurrence of cell death and disease.

Molecules involved in maintaining DA homeostasis have successively become drug targets due to the central role of DA in the pathogenesis of PD. The metabolism of DA *in vivo* is carried out by monoamine oxidase-B (MAO-B) and catechol-O-methyltransferase (COMT) [271–273]. The inhibitors of these enzymes can reduce the degradation of DA and thus play roles in PD treatment.

In detail, the MAO-B inhibitor selegiline has become one of the main drugs in the treatment of PD and is currently approved for use in treatment in China [274]. Recently, rasagiline, a new MAO-B inhibitor, has been approved by the Advisory Committee of the European Medicines Evaluation Agency [274]. DA receptor agonists can bypass the denaturing neurons, directly stimulate the postsynaptic DA receptors, slow down the synthesis of DA, reduce the generation of free radicals, and protect the remaining substantia nigra neurons [275]. Currently, PD treatment is still limited to symptomatic treatment, and the drug target is mostly the production of DA, such as L-DOPa, DA receptor agonists, and the DA-related metabolism enzymes mentioned above [276]. In recent years, both traditional Chinese medicine and acupuncture have achieved good results in the treatment of PD in animal models [277]. They can relieve the motor symptoms of animals with PD and reduce the loss of DAergic neurons in the substantia nigra [277]. These treatments may provide a new therapeutic strategy for PD patients [277, 278].

### 4.4. Acetylcholine

Cholinergic synapses are ubiquitous in the human CNS [279]. Their high density in the thalamus, striatum, limbic system, and neocortex suggests that cholinergic transmission may be critical for memory, learning, attention, and other higher brain functions [208]. The cholinergic system plays an important role in global brain homeostasis and plasticity [280]. Acetylcholine (ACh), the first neurotransmitter to be identified [281], is used by all cholinergic neurons and has a critical important role in the peripheral and CNS [282]. ACh is synthesized from choline and acetyl-coenzyme A (acetyl-CoA) via the enzyme choline acetyltransferase (ChAT) and then transferred by vesicular acetylcholine transporter (VAChT) [283, 284]. When cholinergic neurons depolarize, ACh is released from synaptic vesicles into the synaptic cleft, where it can activate nicotinic receptors (N-receptors) and muscarinic receptors (M-receptors) [208]. ACh in the synaptic cleats is rapidly inactivated by acetylcholinesterase (AChE), thereby releasing choline and acetate [285]. Stimulation of N-receptors present on the membranes of presynaptic neurons in CNS increases the concentration of presynaptic Ca^2+^ [286, 287], which may promote the release of many neurotransmitters, such as ACh, Glu, GABA, DA, serotonin, and norepinephrine [287, 288]. Thus, ACh can influence the strength and fidelity of various synapses and modulate overall CNS neurotransmission [288].

In addition, the cholinergic and glutamatergic systems seem to be interrelated, because the role of ACh in learning and memory seems to be related to the regulation of glutamatergic neurotransmission [221, 289]. Many N-receptor agonists were found, such as nicotine, DMPP (1,1-dimethyl-4-phenylpiperazinium), and cystine [289, 290]. Agonist sensitivity is highly influenced by N-receptor subunit composition [290]. Additionally, curare is the best known antagonist for N-receptors that cannot block CNS nicotinic receptors [291]. M-receptors are widely present in the parasympathetic postganglion fiber-dominated effector cells [208, 292]. When ACh binds to such receptors, it produces a series of parasympathetic terminal excitatory effects [292]. These receptors can also bind to muscarine to produce a similar effect [293, 294]. Atropine, a blocker of these M-receptors, can compete with ACh for M-receptors in the postsynaptic membrane of parasympathetic nerve postganglionic fibers [295], thereby antagonizing muscarinic symptoms and the CNS caused by the excessive acetylcholine stimulation of the postsynaptic membrane. Cholinergic neurotransmission has been implicated in a number of disease states [280, 282]. Defects in cholinergic transmission may affect all aspects of cognition and behavior, including cortical and hippocampal processing of information [296], which was found not only in AD but also in PD, Down syndrome, and ALS [297, 298]. In addition, Huntington's disease seems to be related to the decrease of ChAT activity [297, 298].

Selective injury of cholinergic neurons in the basal forebrains of AD rodent models is reportedly related to increased deposition of A*β* and levels of hyperphosphorylated tau in the hippocampus and cortex [299]. The animal experiments showed that cholinergic depletion promoted A*β* deposition and tau pathology, therefore leading to cognitive impairment [300]. The main therapeutic strategy for AD is to restore cholinergic function through the use of compounds that block the enzymes that break down ACh [301, 302]. Cholinesterase inhibitors (ChEI) are generally considered as the symptomatic treatments for AD [303]. They are a class of drugs that can bind with ChE and inhibit ChE activity [285]; they are also known as anticholinesterase drugs [285, 303]. Their role is to release the ACh accumulated by cholinergic nerve terminals, thereby showing enhanced M-like and N-like effects and activating cholinergic receptors [304]; they are the so-called quasicholinergic drugs [303]. In addition, rivastigmine, donepezil, and galantamine are currently available FDA-approved ChEI drugs used for AD treatment [305]. These drugs have positive effects for only a short period of time (about 1 year to 3 years) and cannot alter disease progression [306].

## 5. Outlook

The synapse is the key structure of the connection among neurons in a neural network and has multiple important physiological functions [6, 7]. Synaptic secretion is involved in several important cellular activities, such as neurotransmitter release, hormone secretion, and natural immunity [8, 309]. The molecular basis of synaptic secretion has fascinated scientists for decades. There are hundreds of proteins involved in regulation, and new ones are still being discovered [57, 310]. Neural communication relies on the tight regulation of synaptic vesicle fusion at nerve endings, which results in neurotransmitter release with strict time and quantum precision [16, 17]. In the resting state, synaptic vesicle fusion is inhibited [19, 311]. When action potentials mediate Ca^2+^ influx to nerve endings, vesicle fusion is induced following the rapid release of neurotransmitters at the millisecond level [311]. These processes are subject to strict regulatory controls that prevent excessive neurotransmitter release and ensure high-fidelity neuronal communication that otherwise leads to disruption of neurotransmission [19, 57, 138, 312].

The coordination of these precise events requires a series of presynaptic proteins [19, 313]. SNARE proteins provide the core fusion mechanism for the energy required for synaptic vesicles to fuse with the plasma membrane [57, 91, 310]. Other biological molecules, such as Synaptotagmin, Munc18, Munc13, CAPS, RIM, Rab, and Complexin, are involved in the regulation of synaptic vesicle secretion in physiological environments [111, 120, 158, 187, 314]. Given this complexity, defects in this mechanism expectedly lead to a range of neurological disorders [2]. There are various neurotransmitters in information communication, including excitatory neurotransmitters and inhibitory neurotransmitters, which play unique roles and jointly regulate neuronal growth and development, synaptogenesis, and synaptic signal transmission [244, 315].

Whether due to genetics, drug abuse, aging process, viral infection, or other reasons, the abnormal communication between neurons may be common to several neuropsychiatric diseases (such as schizophrenia, PD, autism, AD, HD, and depression) [316–318]. Recent studies showed that synaptogenesis disorders can lead to neurological dysfunction [20, 36]; the important pathological changes in several neurodegenerative diseases are the structural changes, the reduction of the number of synapses, and the synaptic dysfunction [36, 203]. It is important to study and elucidate the mechanisms of neurotransmitter release at the molecular level, because understanding these basic mechanisms can better clarify the etiology of neuropsychiatric diseases, which is the key to further understanding the release effect of drugs for disease treatment [318].

**Figure 3:**
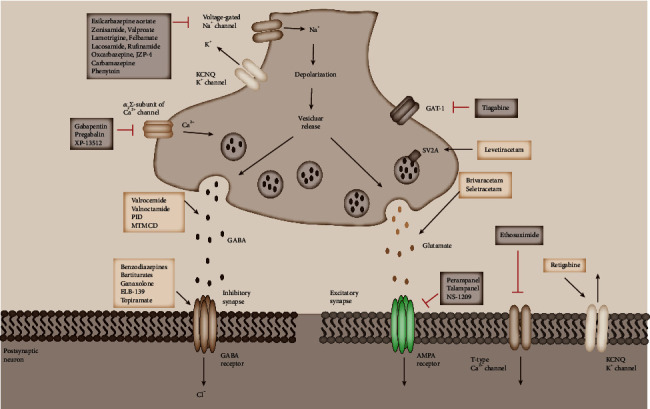
Proposed mechanisms of action of antiepileptic drugs (AEDs) at excitatory and inhibitory synapse. Clinically approved AEDs display a spectrum of mechanisms of action with effects on both inhibitory (left-hand side) and excitatory (right-hand side) nerve terminals. Several synaptic targets of drugs are illustrated, including voltage-gated ion channels (e.g., Na^+^, K^+^, and Ca^2+^), the *α* 2*δ* subunit of the voltage-gated Ca^2+^ channel, vesicular proteins (e.g., SV2A), GABA transporters (GAT-1), GABA receptors, and AMPA receptors.

According to the successfully developed drugs for disease treatment, multiple drugs affecting neurotransmitter transmission act on neurotransmitter receptors, especially presynaptic neurotransmitter receptors (Figure 3 and Table 3) [291, 292]. Some proteins with transport or enzyme functions can also be used as drug targets [291, 303, 319]. Neurotransmitter release mechanisms appear to be relatively poor drug targets, because SNARE proteins, Munc18, Synaptotagmin, and others modulate neurotransmitter release through protein–protein interactions that are difficult to influence with small molecules [320, 321].

Nevertheless, synaptic vesicle protein 2A (SV2A), which is involved in the regulation of neurotransmitter release and vesicle circulation [322], is the action site of the new antiepileptic drug levetiracetam [323, 324]. Currently, 15 anti-PD drugs targeting *α*-synuclein are in the preclinical stage [325]. Therefore, exploring the function and release mechanism of neurotransmitters is of great significance in understanding the role of current drugs and stimulating the development of new drugs.

## 6. Conclusion

Synapses transmit information through synaptic secretion to realize cellular communication. The exocytosis process of vesicles includes tethering, docking, priming, and fusion and mediates the release of transmitters. Damage to any of these steps can lead to functional disorders, further leading to neurodegenerative diseases as well as neurodevelopmental and psychiatric disorders. Important advances have been made in functional models of Ca^2+^-triggered neurotransmitter release mechanisms coregulated by SNARE proteins and other regulatory factors. An in-depth understanding of proteins and their regulatory mechanisms will contribute to a better understanding of neuronal plasticity, as well as diseases caused by cellular communication defects, and have important strategic implications for the prevention and treatment of related diseases and the development of new drugs.

## Figures and Tables

**Table 1:** A list of neurotransmitters types [307, 308]. The types of neurotransmitters, including choline, monoamines, and amino acids and their distribution and functions were shown. PD: Parkinson's disease; AD: Alzheimer's disease; HD: Huntington's disease; ALS: amyotrophic lateral sclerosis; FAD: frontotemporal dementia; VaD: vascular dementia.

Neurotransmitters	Types		Distribution	Function	Diseases
	Choline	Acetylcholine (ACh)	Motor neuron	Affect central function extensively	PD, AD, HD, ALS, FTD, and others
			Tertiary neurons emitted by thalamic afterload, brain stem reticular ascending exciter system	Mainly excitatory and related to learning and memory	AD, HD, ALS, and others
	Monoamines	Dopamine (DA)	The substantia-striatum, limbic system, and nodal-fundal part	An important transmitter of the vertical exoskeleton	PD, AD, HD, ALS, FTD, and others
		Norepinephrine (NE)	Mainly located in the lower brain stem	Excitatory and inhibitory functions are different in different parts of the body	PD, AD, and others
		Serotonin (5-HT)	Concentrated in the raphe nucleus	Associated with sleep, wakefulness, and mood	PD, AD, HD, ALS, FTD, and others
	Amino acids	*γ*-Aminobutyric acid (GABA)	Superficial layer of the cortex, cerebellar cortex	Inhibitory transmitter	AD, VaD, and others
		Glycine	Spinal inhibitory neurons	Inhibitory transmitter	PD, AD, FAD, and others
		Glutamate	Sensory afferent nerve and cerebral cortex	Excitatory transmitter	PD, AD, HD, FAD, and others
	Others	Opioids, brain-gut peptides, NO, and CO can all serve as central neurotransmitters or modulators.			PD, AD, HD, ALS, FTD, and others

**Table 2:** A list of neurotransmitter release processes [19]. The neurotransmitters in the release process, including tethering and docking, priming, and fusion and their definition and functions have been summarized in this table.

Release processes		Definition	Regulatory proteins
	Tethering and docking	The process of vesicle localization on the target membrane. Generally, the distance between the vesicle membrane and the target membrane is about 75~150 nm in tethered state and 5~10 nm in docked state.	SNAREs GTP-binding protein
	Priming	The process of transforming synaptic vesicles into a state with the ability to fuse with the presynaptic membrane of the active zone, which is a rate limiting step in Ca^2+^-dependent exocytosis.	SNAREs, Munc13, Rim, Munc18, CAPS, Snapin, Complexin, Rab3a, Doc2, Syntaphilin Tomosyn, SV2, NSF, SNAPs
	Fusion	Vesicle membrane fuse with presynaptic membrane and release neurotransmitters to synaptic cleft triggered by Ca^2+^ in milliseconds.	SNAREs, Synaptotagmins

**Table 3:** A summary of FDA approved-drugs related to neurotransmitter transmission [256, 262, 276, 285, 302]. The drug name, action mechanisms, application in diseases, and the approval year by the FDA are listed.

Drug name	Mechanism	Application	FDA approval year
Glutamate			
Memantine	NMDA receptor antagonist	AD	2003
Acamprosate	NMDA receptor agonist	The treatment of alcohol dependence	2004
Perampanel	AMPA receptor antagonist	Epilepsy	2012
GABA			
Propofol (Pro)	GABA_A_ receptor agonist	Induction and maintenance of general anesthesia	1989
Baclofen	GABA_B_ receptor agonist	Treats muscle spasms caused by certain conditions (such as multiple sclerosis, spinal cord injury/disease)	2010
Gabapentin	Modulates the action of GAD	Epilepsy	1993
Topiramate	GABA_A_ receptor agonist	Epilepsy	2009
Dopamine			
Selegiline	MAO-B inhibitor	PD	2006
Rasagiline	MAO-B inhibitor	PD	2006
Quetiapine	Dopamine receptor antagonist	AD	1997
Naltrexon/bupropion	Opioid receptor antagonist, dopamine agonist, and NE reuptake inhibitor	Obesity	2014
Clozapine	Dopamine receptor/5-HT2A receptor antagonist	Antipsychotic drugs, mainly for acute and chronic schizophrenia	1990
Risperidone	Dopamine receptor/5-HT2A receptor antagonist	Schizophrenia	2009
Olanzapine	Dopamine receptor/5-HT2A receptor antagonist	Schizophrenia	2009
Aripiprazole	Dopamine receptor/5-HT1A receptor antagonist	Schizophrenia and bipolar disorder	2015
Ziprasidone	Dopamine receptor/5-HT receptor antagonist	Schizophrenia	2001
Rotigotine	Dopamine receptor/5-HT receptor/adrenergic receptor agonist	PD	2007
Acetylcholine			
Rivastigmine	AChE inhibitor	AD	2000
Huperzine A	AChE inhibitor	AD	1999
Donepezil	AChE inhibitor	AD	1996
Galantamine	AChE inhibitor	AD and age-associated memory impairment (AAMI)	2001
Neostigmine	AChE inhibitor	Myasthenia gravis (MG)	2003
Mestinon	AChE inhibitor	MG, obesity, dementia, epilepsy	1955
Atropine	M-receptor antagonist	Antispasmodic agents	2018
Nicotine	N-receptor agonist	Reduces appetite, improves mood, and has some antidepressant properties	1997

## Data Availability

All data generated or analyzed in this study are available from the corresponding author on reasonable request.
